# Relationship between Surgery under General Anesthesia and the Development of Dementia: A Systematic Review and Meta-Analysis

**DOI:** 10.1155/2020/3234013

**Published:** 2020-04-02

**Authors:** Je Jin Lee, Geun Joo Choi, Hyun Kang, Chong Wha Baek, Yong Hun Jung, Hwa Yong Shin, Yong Hee Park, Young Cheol Woo

**Affiliations:** ^1^Department of Anesthesiology and Pain Medicine, Chung-Ang University Hospital, Seoul 06973, Republic of Korea; ^2^Department of Anesthesiology and Pain Medicine, Chung-Ang University College of Medicine, Seoul 06974, Republic of Korea

## Abstract

**Objective:**

To investigate the association between exposure to general anesthesia and the development of Alzheimer's disease (AD) and dementia by reviewing and integrating the evidence from epidemiological studies published to date.

**Methods:**

We searched MEDLINE, EMBASE, and Google Scholar to identify all relevant articles up to April 2018 reporting the risk of AD/dementia following exposure to general anesthesia and finally updated in February 2020. We included patients older than 60 or 65 years who had not been diagnosed with dementia or AD before the study period. The overall pooled effect size (ES) was evaluated with a random-effect model. Subgroup analyses were conducted and possibility of publication bias was assessed.

**Results:**

A total of 23 studies with 412253 patients were included in our analysis. A statistically significant positive association between exposure to general anesthesia and the occurrence of AD was detected in the overall analysis (pooled ES = 1.11, 95%confidence interval = 1.07–1.15), but with substantial heterogeneity (*p*_*χ*_^2^ < 0.001, *I*^2^ = 79.4). Although the overall analysis revealed a significant association, the results of the subgroup analyses were inconsistent, and the possibility of publication bias was detected.

**Conclusion:**

s. This meta-analysis demonstrated a significant positive association between general anesthesia and AD. However, considering other results, our meta-analysis must be interpreted with caution. Particularly, it should be considered that it was nearly impossible to discriminate the influence of general anesthesia from the effect of surgery itself on the development of AD. Further, large-scale studies devised to reduce the risk of bias are needed to elucidate the evidence of association between general anesthesia and AD. *Trial registration*. PROSPERO International prospective register of systematic reviews CRD42017073790.

## 1. Introduction

With the development of medical technology and healthcare systems, life expectancy has increased worldwide. Accordingly, the number of surgeries performed in elderly patients has also been increasing [1]. However, the high morbidity and mortality following surgery are major concerns to the elderly themselves, surgeons, anesthesiologists, and policymakers.

Dementia is a progressive neurodegenerative disease characterized by multiple cognitive impairments that represent a decline from one's previous level of functioning. The global prevalence rate of dementia in people over the age of 60 ranges from 5–7%, and increases rapidly with age, to 20% in people over the age of 85 years [2]. The Global Burden of Disease Study 2015 reported that the global prevalence of dementia has increased from 21.7 million in 1990 to 46.0 million in 2015 [3], and it is expected to be more than 100 million by 2050 [4]. Dementia has become a substantial public health concern because of its progressive, irreversible course and high prevalence in the elderly [5].

Alzheimer's disease (AD) is the most common cause of dementia, accounting for approximately 60–80% of dementia cases [6]. The pathophysiology of AD is still unclear but is mainly associated with accumulation of extracellular beta-amyloid plaques and intracellular neurofibrillary tangles, which lead to neuronal cell death and degeneration [6]. There is no single method for the definitive diagnosis of AD. The prevailing guidelines include the Diagnostic and Statistical Manual of Mental Disorders (DSM) and the National Institute of Neurological and Communicative Disorders and Stroke and the Alzheimer's Disease and Related Disorders Association (NINCDS-ADRDA) criteria [7]. However, some physicians diagnose AD based on their clinical knowledge and not on these guidelines [8–13].

Previous studies have suggested advanced age, female sex, family history of AD, cardiovascular disease, head trauma, depression, and lower educational level as potential risk factors [6, 12, 14, 15]. Despite these reports, the precise causes and risk factors for AD still remain unclear because of its multifactorial and complex features. Recently, it has been suggested that previous general anesthesia exposure may act as a risk factor for AD in the elderly [16, 17]. Although some experimental data obtained in cells and animal models have suggested a significant association between general anesthetics, especially volatile agents [18], and AD, evidence from human studies is controversial.

There have been several attempts to integrate the evidence from various epidemiological studies, such as the reanalysis of eight case-control studies [19] and two meta-analyses [20, 21]. Even though they consistently showed no evidence of an association between general anesthesia and dementia/AD, an updated meta-analysis is needed because the previous meta-analyses incorporated non-peer-reviewed articles and studies using excessively broad outcome measures. Moreover, two of the previous meta-analyses [19, 21] included only case-control studies, which are prone to bias. We critically reviewed and synthesized the current evidence to determine the association between administration of general anesthesia and development of AD and to verify whether general anesthesia acts as a risk factor for AD in the elderly.

## 2. Methods

The protocol for this review has been registered in the PROSPERO network (registration number: CRD42017073790) and published in a peer-reviewed journal [22]. This systematic review and meta-analysis of the association between general anesthesia and the development of AD were performed according to the Meta-analysis of Observational Studies in Epidemiology (MOOSE) guidelines [23] and reported according to the guidelines of Preferred Reporting Items for Systematic reviews and Meta-Analysis (PRISMA) [24].

### 2.1. Search Strategy

A search was performed by two different investigators independently in MEDLINE, EMBASE, and Google Scholar for articles up to April 2018 using search terms related to AD, dementia, and general anesthesia and updated in February 2020. The search terms used in MEDLINE and EMBASE are presented in [Supplementary-material supplementary-material-1]. In order to identify all relevant articles, we scanned the reference lists of the original papers until no further relevant references could be found. No language or date restrictions were applied.

### 2.2. Selection Criteria

Our selection criteria are as follows.

#### 2.2.1. Study Design

Peer-reviewed cohort and case-control studies including nested case-control studies were eligible for inclusion. We excluded data from proceedings, letters to the editor, posters, commentaries, laboratory science studies, and any other nonrelevant studies.

#### 2.2.2. Population

Inclusion criteria for study populations were as follows: (1) the elderly (defined as more than 60 or 65 years old) from all countries and (2) those who had not been diagnosed with dementia or AD before the beginning of the study period. If the study's definition of the elderly was other than being older than 60 or 65 years of age, an attempt was made to contact the study authors to obtain the relevant information. When unsuccessful, we performed a pooled analysis including the data of that study first, and then, we performed a sensitivity analysis excluding the data. No restrictions were applied in terms of sex, race/ethnicity, or socioeconomic status.

#### 2.2.3. Exposure

Exposure to general anesthesia for surgery, usually using inhalation anesthetics, was included. Intravenous anesthesia, spinal anesthesia, epidural anesthesia, and regional anesthesia were excluded. If an article reported on general anesthesia including intravenous, spinal, epidural, or regional anesthesia along with inhalation anesthesia, we tried to contact the study authors to obtain information on general anesthesia using inhalation anesthetics. When unsuccessful, we first analyzed the data on general anesthesia, including intravenous, spinal, epidural, or regional anesthesia, and then we performed sensitivity analysis excluding the data. The source of exposure assessment was also collected.

#### 2.2.4. Comparison

Comparison groups included individuals with no history of general anesthesia. If a study only reported previous anesthesia history during the study period, we tried to contact the study authors for further information about previous anesthesia history prior to the study period. When unsuccessful, the reported information was used for our analysis. If a study investigated the associations of AD and general anesthesia using two or more comparison groups and reported each outcome separately, pooled estimates of associations for these groups were calculated and used for analysis.

#### 2.2.5. Outcome Measures

To cover as many AD cases as possible, we included not only AD cases but also dementia cases, of which AD cases comprise the largest portion, diagnosed by standard criteria such as the DSM or clinically diagnosed by a professional physician. Studies reporting effect size (ES) as odds ratio (OR), relative risk (RR), or hazard ratio (HR) of dementia/AD to general anesthesia exposure were included. In some studies, which reported only the number of individuals with and without AD instead of ES, we obtained the ES by calculation from the data provided. When the study reported only dementia cases without distinguishing AD [25–28], we attempted to contact the study authors to obtain the classified data. If the attempt was unsuccessful, we conducted a pooled analysis and examined the difference by subgroup analysis.

### 2.3. Study Selection

Reference lists obtained as described above were imported into Endnote software (Thompson Reuters, CA, USA), and duplicate articles were removed. The titles and abstracts identified through the search strategy were scanned independently by two investigators. To minimize data duplication as a result of multiple reporting, papers from the same author were compared. For reports determined to be eligible based on the title or abstract, the full paper was retrieved. Potentially relevant studies chosen by at least one investigator were retrieved and evaluated in full-text versions. Articles meeting the inclusion criteria were assessed separately by two investigators, and any discrepancies were resolved through discussion. In cases where agreement could not be reached, the disputes were resolved with the help of a third investigator.

### 2.4. Data Extraction

Using a standardized extraction form, the following data were extracted independently by two reviewers: study name (along with the name of the first author and year of publication); region where the study was conducted; study design; source from which subjects were selected; age of subjects; exposure definition; method of data collection (self-reported vs. medical records); outcome definition; ES such as OR, RR, and HR with 95% confidence intervals (CIs); methods for controlling covariates and the confounding variables controlled for; number of cases/controls or cohort groups; and total number of participants. If information was missing, an attempt was made to contact the study authors to obtain the relevant information. When unsuccessful, missing information was calculated if possible from the relevant data in the study. As the ES was not reported or needed to be integrated because of the multiple groups, it was calculated in six studies [8, 12, 13, 29–31] using the relevant data. The reference lists were divided in half, and two reviewers completed the data extraction for each half of the list. Then, data extraction forms were cross-checked to verify the accuracy and consistency of extracted data.

### 2.5. Study Quality Assessment

The quality of the studies was independently assessed by two investigators using the Risk of Bias Assessment Tool for Nonrandomized Studies (RoBANS) [32]. The quality of each study was evaluated according to the following six domains: the selection of participants, confounding variables, the measurement of exposure, the blinding of the outcome assessments, incomplete outcome data, and selective outcome reporting. The methodology of each study was graded as “high,” “low,” or “unclear” to indicate high risk of bias, low risk of bias, and unclear risk of bias. Any discrepancies were resolved through discussion. If an agreement could not be reached, the dispute was resolved with the help of a third investigator.

### 2.6. Statistical Analysis

All statistical analyses were performed using Stata SE version 15.0 (StataCorp, College Station, TX).

#### 2.6.1. Data Synthesis

Overall pooled ES and its corresponding 95% CI and 95% prediction interval were computed. Between-study heterogeneity was assessed using the Cochran's *Q* and Higgins's *I*^2^ statistics [33]. A *p* value of <0.10 for the *χ*^2^ statistic or an *I*^2^ greater than 50% was considered as showing heterogeneity, and data were analyzed using the Mantel–Haenszel random-effect model. Otherwise, we applied the Mantel–Haenszel fixed-effect model [34].

#### 2.6.2. Subgroup Analysis

Subgroup analysis was carried out based on study design (case-control vs. cohort study), region of the study population, exposure assessment (self-reported vs. medical record), outcome definition (AD vs. dementia), and method of case ascertainment (standard criteria vs. clinical diagnosis).

#### 2.6.3. Sensitivity Analysis

We conducted sensitivity analyses to evaluate the influence of individual studies on the overall effect estimate by excluding one study at a time from the analysis.

#### 2.6.4. Publication Bias

Publication bias was assessed by using contour-enhanced funnel plots and Egger's test [35]. An asymmetric contour-enhanced funnel plot or a *p* value < 0.1 from Egger's test was considered to indicate the presence of publication bias. If publication bias was detected, trim and fill analysis was performed.

## 3. Results

### 3.1. Study Selection and Characteristics

**Figure 1:**
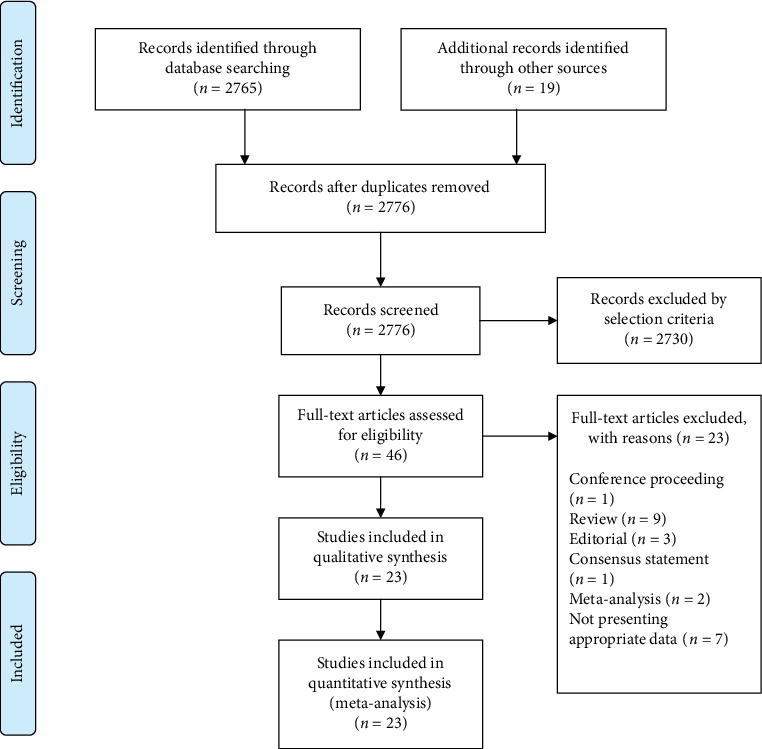
PRISMA flow diagram of literature search and selection.

A total of 2784 articles were obtained after searching the databases and references and through the manual search (Figure 1). After excluding the duplicates (*n* = 8), we reviewed the remaining articles (*n* = 2776); 2730 of the articles did not meet the selection criteria. The remaining 46 articles were selected for review of their whole content. The kappa value for selecting articles between the two reviewers was 0.756.

Of the 46 studies selected for review of their whole content, 23 were excluded for the following reasons: One was a conference proceeding [36], 9 were reviews [17, 37–44], 3 were editorials [45–47], 1 was a consensus statement [48], 2 were meta-analyses [19, 20], and 7 did not present the appropriate data [49–55]. Some of the studies required additional consultation among the reviewers to determine whether they should be included [8, 9]. One study was about comparing the risk of AD following coronary artery bypass graft (CABG) and percutaneous transluminal coronary angioplasty (PTCA) [9]. Considering that CABG is a surgery requiring general anesthesia and PTCA is not, we decided to include this study in our pooled analysis. Another study investigated whether spine surgery contributes to the development of AD [8]. There were no details about the anesthesia procedure. However, the spine surgery ranged from discectomy to complex spine fusion procedures, all typically performed under general anesthesia. Therefore, we also included this study in our research. Therefore, 23 studies involving a total of 412253 patients were included in our analysis. To obtain more relevant information than reported, we tried to contact some authors of the included studies. Among the contacted authors, one [27] provided additional data about the study population sufficient for our complete analysis.

The study characteristics are summarized in Table 1. The studies comprised 6 cohort studies (2 prospective [27, 30] and 4 retrospective [8, 9, 26, 31]) and 17 case-control studies [10–15, 25, 28, 29, 56–63], including 3 nested case-control studies [25, 28, 59]. The studies were conducted in America [8–11, 13, 30, 56, 57, 59, 60], Australia [58], Asia [15, 25–27, 63], and Europe [12, 14, 28, 29, 31, 61, 62]. 14 studies reported the risk of AD [8–15, 29, 56–58, 60], 6 studies reported the risk of all-cause dementia [25–28, 31, 62], and 3 studies reported the risk of both AD and dementia [30, 59, 63]. Twelve of the studies used medical records as a means of exposure assessment [8–11, 25–27, 29, 31, 59, 62, 63] and the others used self- or surrogate-reported data by interview or questionnaire [12–15, 28, 30, 56–58, 60, 61]. Diagnosis for AD or dementia was established using standard criteria such as the DSM-IV and NINCDS-ADRDA [14, 30, 59, 63], DSM-III and NINCDS-ADRDA [56, 57], NINCDS-ADRDA [29, 58, 60], NINCDS-ADRDA and International Classification of Diseases (ICD)-10 [15], Automated Geriatric Examination for Computer Assisted Taxonomy (AGECAT) algorithm [28], ICD-9 [8, 9, 25, 26], ICD-10 [27, 31], or the physician's own clinical diagnosis [10–13].

### 3.2. Study Quality Assessment

Overall risks of bias evaluated using the RoBANS are shown in Table 2. One study was assessed as having unclear risk of bias in selection of participants because of the lack of mention about the process of evaluating the cognitive function of controls with Parkinson's disease and nondegenerative neurological disease or verifying the absence of dementia [29]. We rated the studies which used self- or surrogate-reported data as “unclear” in the domain of measurement of exposure. As we could not find any protocol from the studies to compare, we graded every study as having unclear risk of bias in selective outcome reporting.

### 3.3. Meta-Analysis of Overall Studies

**Figure 2:**
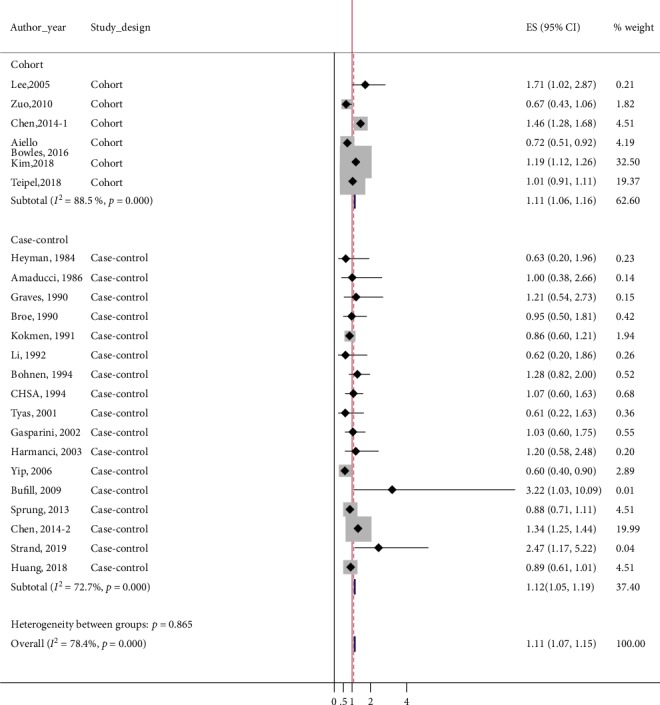
Forest plot for overall studies showing the risk of Alzheimer's disease (AD) following general anesthesia. An effect size of 1 (red vertical line) indicates no effect of general anesthesia in development of AD. The gray-colored box of each study means the weight of the study data. Overall pooled effect size showed significantly increased risk of AD following general anesthesia. Above 6 studies are subgroup of cohort studies, and following 17 studies are subgroup of case-control studies. Both of them showed significantly high risk of AD following general anesthesia.

After pooling all available data, we observed a significant positive association between the risk of AD and general anesthesia exposure (overall pooled ES = 1.11, 95%CI = 1.07–1.15). However, results of the *Q* test and *I*^2^ statistics suggested substantial heterogeneity (*p*_*χ*_^2^ < 0.001, *I*^2^ = 79.4) (Table 3 and Figure 2). 95% prediction interval for overall studies was 0.98-1.21, including 1.00, therefore implying any future study could change the significance of association between general anesthesia and AD. Sensitivity analysis by excluding the study reports unadjusted ES [8] did not show any change in significance of results (overall pooled ES = 1.12, 95%CI = 1.08–1.16).

As the heterogeneity for ES was considerable, metaregression was conducted to determine the origin of heterogeneity. According to metaregression, the study design, region, exposure assessment, case ascertainment, definition of dementia or AD, and year of publication were not likely to be a source of heterogeneity (Table 4).

### 3.4. Subgroup Analyses

The results of the subgroup analyses are displayed in Table 3. In subgroup analysis according to study design, both cohort studies (pooled ES = 1.11, 95%CI = 1.06–1.16; *p*_*χ*_^2^ < 0.001, *I*^2^ = 88.5) and case-control studies (pooled ES = 1.15, 95%CI = 1.03–1.17; *p*_*χ*_^2^ < 0.001, *I*^2^ = 74.8) showed a significantly higher risk of AD in people who had been exposed to general anesthesia, but with substantial heterogeneity (Figure 2). 95% prediction interval was 0.93-1.27 and 0.81-1.20 in cohort studies and case-control studies, suggesting any further study could alter the significance of association between general anesthesia and AD.

**Figure 3:**
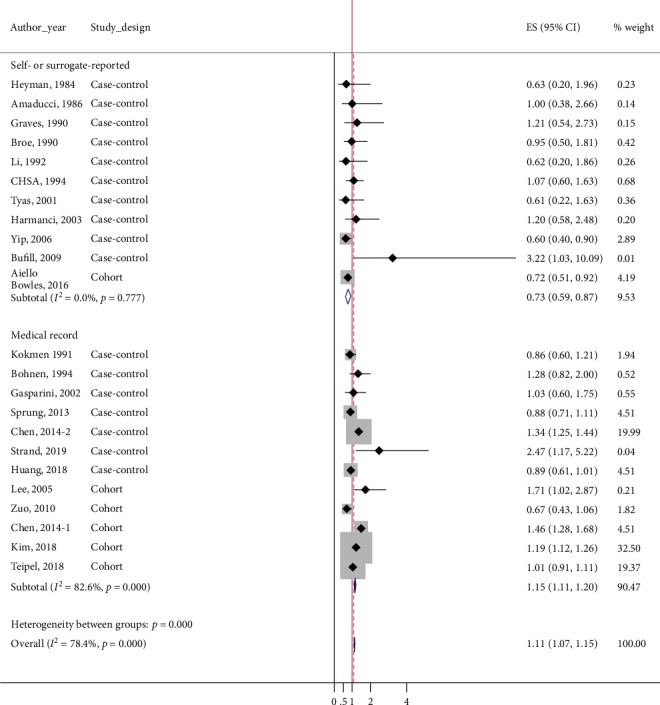
Forest plot for subgroups based on exposure assessment: self- or surrogate-reported and medical record. The risk of dementia following general anesthesia was significantly high in medical record subgroup, but it was significantly low in self or surrogate-reported subgroup.

Subgroup analysis based on the exposure assessment method showed conflicting results. In subgroup of studies using medical records, the risk of AD was significantly high in general anesthesia-exposed patients (pooled ES = 1.15, 95%CI = 1.11–1.20; *p*_*χ*_^2^ < 0.001, *I*^2^ = 82.6). However, negative association was detected from the subgroup of studies based on self- or surrogate-reported data (pooled ES = 0.73, 95%CI = 0.59–0.87; *p*_*χ*_^2^ = 0.777, *I*^2^ = 0.0) (Figure 3).

**Figure 4:**
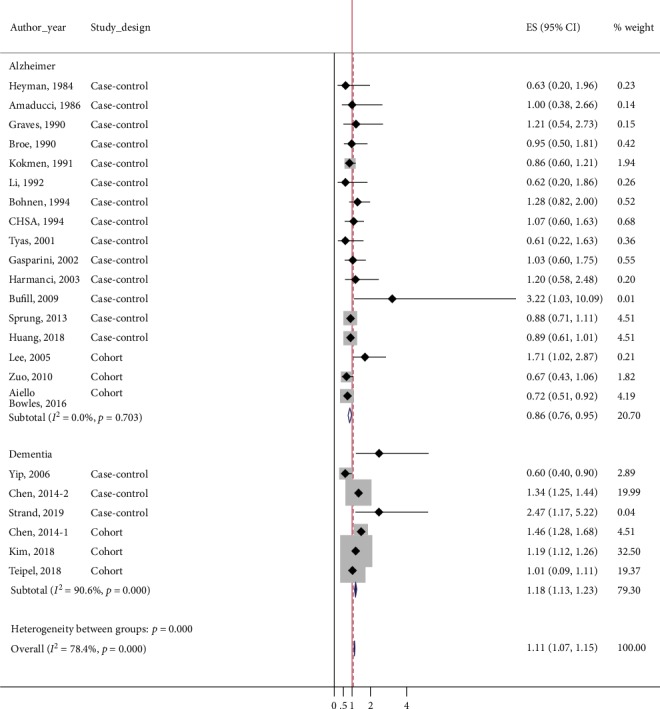
Forest plot for subgroups according to outcome definition: Alzheimer's disease and all-cause dementia. An increased risk of dementia following general anesthesia was detected among all-cause dementia group, but with considerable heterogeneity. However, decreased risk of AD with general anesthesia exposure was detected among the studies with outcome definition limited to only Alzheimer's disease.

In the subgroup analysis according to the definition of outcome, general anesthesia was associated with an increased risk of dementia when the outcome was defined as all-cause dementia (pooled ES = 1.18, 95%CI = 1.13–1.23; *p*_*χ*_^2^ < 0.001, *I*^2^ = 90.6), but with considerable heterogeneity. However, the pooled ES among the studies with an outcome definition limited to only AD suggested decreased risk of AD in patients with general anesthesia exposure (pooled ES = 0.86, 95%CI = 0.76–0.95; *p*_*χ*_^2^ = 0.703, *I*^2^ = 0.0) (Figure 4).

**Figure 5:**
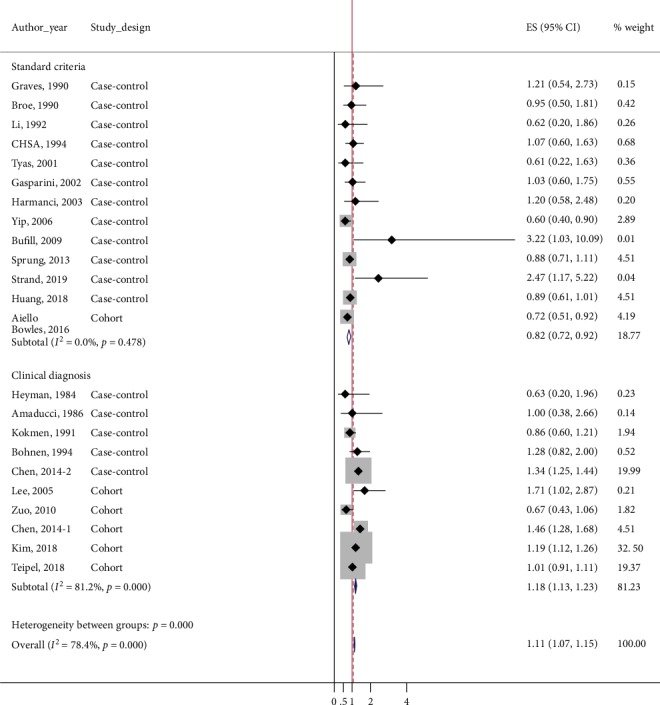
Forest plots for subgroups based on case ascertainment: standard criteria and clinical diagnosis. Whereas the pooled effect size of the studies, which clinically diagnosed dementia, showed a significant positive association between general anesthesia and dementia, inversely negative association was observed among the studies using standard diagnostic criteria of dementia.

Subgroup analysis of the studies that used clinical diagnosis showed a significant positive association between general anesthesia and AD (pooled ES = 1.18, 95%CI = 1.13–1.23; *p*_*χ*_^2^ < 0.001, *I*^2^ = 81.2). In contrast, an inversely negative association was observed among the studies using standard diagnostic criteria (pooled ES = 0.82, 95%CI = 0.72–0.92; *p*_*χ*_^2^ = 0.478, *I*^2^ = 0.0) (Figure 5).

### 3.5. Sensitivity Analysis

Sensitivity analysis was performed by excluding one study at a time; no change in statistical significance occurred.

### 3.6. Publication Bias

**Figure 6:**
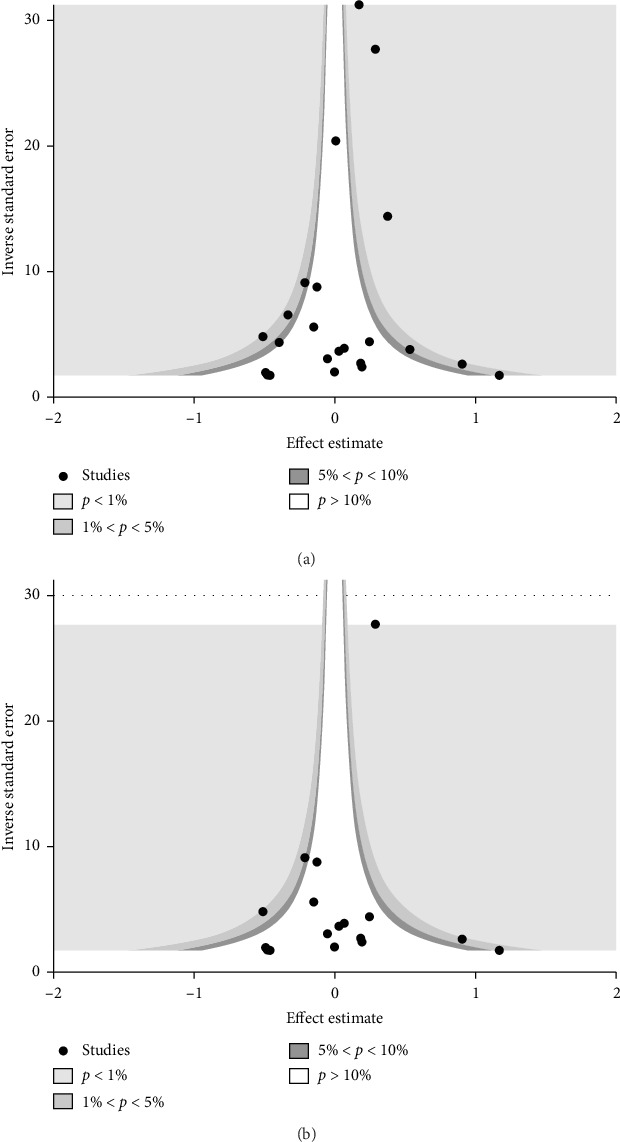
Contour-enhanced funnel plots of total studies (a) and case-control studies (b). Asymmetry was observed in funnel plots of both total studies and case-control studies.

Contour-enhanced funnel plots were asymmetric for overall studies and case-control studies (Figure 6). Egger's test also showed significant results for overall studies (Coef = −0.91, 95%CI = −1.99–0.24, *p* = 0.096) and case-control studies (Coef = −1.02, 95%CI = −2.22–0.17, *p* = 0.088) (Table 3). Therefore, to evaluate the influence of publication bias for these studies, trim and fill analyses were performed. After trim and fill analyses, significance disappeared in overall studies (pooled ES = 1.09, 95%CI = 0.94–1.27) and case-control studies (pooled ES = 1.07, 95%CI = 0.87–1.32) (Table 3).

## 4. Discussion

The effect of general anesthetics, especially inhalation agents, on neurocognitive function is currently controversial; it is unknown whether general anesthetics are neurotoxic or neuroprotective [64]. Regarding neurotoxicity, numerous in vitro and in vivo studies with cells, tissues, animals, and biomarkers have suggested that volatile anesthetics may contribute to the neuropathogenesis of AD. However, evidence from human studies is very weak. This systematic review and meta-analysis were designed to investigate the association between general anesthesia and the risk of AD in human studies. By integrating the conflicting evidence documented to date, we detected a statistically significant association between exposure to general anesthesia and the occurrence of AD. This significant positive association was consistent in both cohort studies and case-control studies. In addition, the result of subgroup analysis of studies based on medical records, a trustworthy data source, supported the significant positive association between general anesthesia and AD.

Notably, this outcome was contrary to the results of two previous meta-analyses [20, 21] and a reanalysis [19], which reported no evidence of a significant relationship between general anesthesia and dementia. The differences between our investigation and the previous studies may explain the contradictory results. First, our meta-analysis includes recently published literature. The meta-analysis published in 2011 [21] included only case-control studies, which are susceptible to bias. Although another meta-analysis of epidemiological studies up to April 2017 was conducted lately [20], a new large-scale population-based prospective cohort study was published in April 2018 [27]. Including this study, we have evaluated six more relevant studies [26, 27, 31, 60, 62, 63] than the previous meta-analysis. Second, there were some differences in study selection between our analysis and the others. We included only peer-reviewed cohort and case-control studies to ensure the reliability of the evidence. Therefore, some of the studies which had been included in the previous meta-analyses were excluded because they were non-peer-reviewed articles [36, 44]. Several other studies have been excluded from our analysis because they did not meet our inclusion criteria: One study was about the risk of AD following occupational exposure to anesthetic gases [54], one included not only dementia but also mild cognitive impairment as outcomes [53], and one had a completely different study purpose than ours [55].

Given the various designs and methods used in the involved studies, we performed subgroup analyses to better understand the results of our meta-analysis. In the subgroup analysis based on exposure assessment, an increased risk of AD following exposure to general anesthesia was observed in studies using medical records. Because medical records are more objective and reliable data source than self-reported data collected through interview, this result enhances the validity of the positive association between general anesthesia and AD observed in overall analysis. In addition, as shown in Figure 3, total weight of the studies based on medical record was about 90%. Thus, the positive association detected from the studies based on medical records seems to have contributed significantly to the overall outcome. Although a low risk of AD was observed in the subgroup of studies based on self- or surrogate-reported data, it might be questionable since interview-based data that relies on person's memory is prone to involve recall bias. Elderly subjects, especially those who have memory decline, and their family members may have difficulty in remembering the details of medical history.

However, according to the subgroup analysis based on case ascertainment, a negative association was observed in studies using standard criteria. Standard criteria are usually considered more reliable diagnostic tool because it is less likely to involve physician's subjectivity compared to clinical diagnosis. This finding suggests that the results of the overall analysis must be interpreted with caution.

Considering that AD cases comprise the largest portion of dementia cases (60–80% in one study) [6], we included studies on all-cause dementia in order to collect as many AD cases as possible. In subgroup analysis according to outcome definition, a stronger positive association was detected among the studies that defined cases as all-cause dementia, compared to the total analysis. On the other hand, studies that collected only AD cases showed a negative association between previous anesthesia exposure and AD. In the current diagnostic criteria, AD is diagnosed by excluding evidence of vascular dementia. Recently, however, the concept of mixed dementia (mixed vascular–Alzheimer dementia) has been emerging [65], and currently used diagnostic criteria may yield lower incidence rate of AD than the actual value. Recent studies have suggested that about half of older adults with dementia have pathological evidence of more than one cause of dementia [6]. Besides, under certain circumstances, surgery and anesthesia are risk factors for cerebral ischemia [66]. These factors may have increased the differences between the risk of AD and all-cause dementia including AD and mixed vascular–Alzheimer dementia following surgery under general anesthesia. Additionally, these factors might have contributed to the statistically negative association between general anesthesia and AD observed in studies using standard diagnostic criteria.

One of the factors important in outcome assessment is lag time, which means the latency period before the diagnosis of disease. In studies without including the lag time, the patients with incidental dementia not severe enough to be diagnosed may be misinterpreted as having dementia caused by general anesthesia exposure. As highlighted in the meta-analysis conducted to verify the association between dementia and benzodiazepine which was published in 2018 [67], analysis considering lag time is important enough to influence the statistical significance of the findings. However, among the 23 studies included in our analysis, there was only one study introduced the lag time to their methodology [31]. Although the cohort studies excluded the patients with diagnosis of dementia or AD prior to enrollment, it was difficult to find the details about ensuring that dementia or AD was not present before the exposure of general anesthesia in the case-control studies. Thus, even though the positive association was detected from our findings, it is insufficient to verify the causality between general anesthesia and AD because of the uncertainty of time sequence and the lack of considering lag time.

Therefore, although a significant association between general anesthesia exposure and AD was observed, the results of this meta-analysis should be interpreted with caution. The possibility of publication bias was demonstrated by contour-enhanced funnel plots and Egger's test. The statistical significance disappeared after trim and fill analysis. In addition, the 95% prediction interval computed from overall studies implied that any future study could alter the statistical significance of association between general anesthesia and AD. These findings support the need for prudence to properly interpret the results of this meta-analysis.

Our study has some limitations. First, substantial heterogeneity was observed among the included studies. Although various approaches were attempted to reveal the origin of heterogeneity, they were not successful. Second, because of insufficient details on the context of anesthesia including number of exposure, the agent used for induction and maintenance, dose and duration of exposure, and intraoperative events or perioperative complications such as hemodynamic instability and hypoxia, we could not conduct analysis adjusting for these variables. Moreover, surgery itself might contribute to an increased risk of AD. Although there are no data on the risk of AD, high-risk surgeries such as cardiac surgery have been reported as raising the risk of cognitive impairment such as delirium and postoperative cognitive dysfunction [42, 68]. However, it is nearly impossible to analyze the effect of anesthesia and surgery separately, since anesthesia is not performed alone. Third, contour-enhanced funnel plots and Egger's test suggested the possibility of publication bias derived from studies that are not published in the current literature because of null results or small sample size.

According to the limitations discussed, additional well-designed studies are necessary to clarify the relationship between general anesthesia and dementia or AD. Future studies are recommended to be conducted with the following considerations: large-scale study with adequate statistical power; prospective cohort studies with long-term follow-up including lag time, ensuring the subjects free of dementia before exposure of anesthesia; using reliable data source such as medical records; reporting sufficient details on the characteristics of exposure such as number of exposure, agents, and doses used; adequate adjustments for confounding variables; and outcome assessment with standard diagnostic tool of dementia. Recent review published by American scholars reported a wide variety of diagnostic methods for dementia used in large cohort studies, while emphasizing the need for the development of well-described and reproducible methods for diagnosing dementia in epidemiologic studies [69]. We believe that following standardized and reproducible process will reduce the heterogeneity and enhance the comparability of studies.

Despite the limitations above, our systematic review and meta-analysis demonstrate the strengths of a rigorous methodology based on a published, preplanned protocol to provide evidence of the relationship between general anesthesia exposure and risk of AD. Furthermore, our study has a value of suggesting general anesthesia exposure as a potential risk factor of AD and raising the necessity of further related studies for better management of the elderly. Recently, there was a meta-analysis reported the significantly high mortality rates of the patients with dementia after undergoing hip fracture surgery, emphasizing the importance of perioperative care for dementia [70]. Although dementia is not a fatal condition, it can seriously degrade one's quality of life and even deteriorate the prognosis after surgery. In an aging society, further research for better understanding of the risk factors of dementia and perioperative managing of dementia patients are becoming valuable [71, 72].

## 5. Conclusion

In conclusion, we observed a significant association between exposure to general anesthesia and an increased risk of AD. However, considering the substantial heterogeneity, evidence of publication bias, and inconsistent results of the subgroup analyses, the results of our meta-analysis should be interpreted with caution. Moreover, it was nearly impossible to discriminate the influence of general anesthesia from the effect of surgery itself on the development of AD. Further, large-scale prospective cohort studies designed to reduce the risk of bias considering lag time, using standardized methods and reliable data with adequate adjustments of confounding factors, are needed to elucidate the evidence of an association between general anesthesia and AD.

## Figures and Tables

**Table 1:** Study characteristics.

Study (1st author, year) Region	Study design	Study population	Source of data extraction	Method of exposure assessment	Outcome definition	Lag time; adjusted or matched covariates	Group definition	*N*	Statistics (95% CI)
Cohort study									
Kim, 2018 [27] South Korea	PC	From the South Korean NHIS-NSC database. Age ≥ 50 free of dementia	Patient records on NHIS-NSC database files between 2002 and 2013	GA operation codes in the NHIS-NSCdatabase	Dementia (clinical diagnosis using ICD-10 codes and history of dementia medication)	Did not included lag time; adjusted for gender, age group, health security system, health care visit frequency, and Charlson comorbidity index			HR
							GA group	44954	1.285 (1.214–1.361)
							Age 60–69	17387	1.216 (1.118–1.322)
							Age > 69	5655	1.162 (1.059–1.276)
							Unexposed group	174469	1.000
							Age 60–69	57867	1.000
							Age> 69	41461	1.000
Teipel, 2018 [31] German	RC	From the German statutory health insurance database. Age ≥ 65	Medical records from AOK	History of joint replacement surgery	Dementia (clinical diagnosis using ICD-10 codes)	Included lag time; adjusted for cerebrovascular risk factors, age, sex, the presence of delirium, and regular prescription of sedative or analgesic drugs (SAD)	No surgery	154604	Calculated HR 3.55 (3.13–4.03)
							Quarter 0	10563	
							1-3 quarter		0.95 (0.82–1.11)
							4-6 quarter		0.83 (0.70–0.97)
							≥7 quarter		0.91 (0.83–0.99)
Aiello Bowles, 2016 [30] Washington, USA	PC	Adult Changes in Thought (ACT) cohort which was randomly selected from members of Group Health (GH). Age ≥ 65 free of dementia	Self-reported data through interview at baseline and follow-upstudy visits	Self-reported anesthesia data (reviewed by anesthesiologist)	Dementia (DSM-IV) AD (possible or probable AD by NINCDS-ADRDA)	Did not included lag time; adjusted for ACT study cohort, age, age at study entry, sex, education, hypertension, diabetes mellitus, smoking, stroke, coronary heart disease, exercise, self-rated health, body mass index, depression, Parkinson's disease, Charlson comorbidity index, and difficulty with activities of daily living.	High-risk surgery with GA	248	HR (dementia/AD) 0.86 (0.58–1.28) 0.95 (0.61–1.49)
							Other surgery with GA	3363	0.63 (0.46–0.85) 0.65 (0.46–0.93)
							Other surgery with neuraxial anesthesia	123	0.49 (0.26–0.90) 0.62 (0.32–1.19)
							No anesthesia group	254	1.00 1.00
Chen, 2014-1 [26] Taiwan	RC	LHID (a subset of the Taiwan NHIRD). Age ≥ 50 without history of cancer, dementia, parkinsonism, stroke, and brain surgery	Records from the LHID between 2004 and 2007	Record of anesthesia from the LHID	Dementia (clinical diagnosis using ICD-9-CM)	Did not included lag time; matched for exact age and sex. Cox regressions adjusted for hypertension, hyperlipidemia, depression, and Charlson index.			HR
							Anesthesia group	24901	1.75 (1.59–1.92)
							General	13715	1.46 (1.28–1.68)
							IV or IM	1686	1.60 (1.11–2.30)
							Regional	8777	1.80 (1.57–2.07)
							Control group (4 or 5 patients selected for each person in anesthesia group)	110972	1.00
Zuo, 2010 [8] Virginia, USA	RC	From the CDR containing deidentified information of inpatients and outpatients in the University of Virginia Health System.	Medical records from the CDR	Record of spine surgery under GA	AD (clinical diagnosis using ICD-9-CM)	Did not included lag time; none	Spine surgery group (from discectomy to fusion between 1992 and 2004)	2881 (age ≥ 60)	Calculated OR using number of patients 0.67 (0.43–1.06)
							No surgery group	6157 (age ≥ 60)	1.00
Lee, 2005 [9] USA	RC	Veterans Affairs (VA) patients undergoing CABG or PTCA between October 1996 and September 1997. Age ≥ 55 without AD	VA administrative databases of inpatient and outpatient encounters	History of CABG (not mentioned about GA, but necessary)	AD (clinical diagnosis using ICD-9)	Did not included lag time; adjusted for age, number of surgeries, number of diagnoses, and length of stay for index hospitalization.	CABG group (including patients who had both CABG and PTCA)	5216	HR 1.71 (1.02–2.87)
							PTCA group	3954	1.00
Case-control studies									
Strand, 2019 [62] Sweden	CC	Case: Swedish Dementia Quality Registry, diagnoses of AD, late-onset AD, early-onset AD, and mixed Alzheimer's and vascular dementia in the county of Östergötland from May 2007 to April 2012 Control: selected from the Statistics Sweden	Medical records	Medical record of prior GA with gas	Dementia (diagnoses of AD, late-onset AD, early-onset AD, and mixed Alzheimer's and vascular dementia from the dementia registry)	Did not included lag time; adjusted for age category, sex, hypotension under anesthesia, total time anesthesia, and number of exposures of anesthesia.	Cases	457	OR 2.47 (1.17–5.22)
							Controls	420	1.00
Huang, 2018 [63] China	CC	Case: residents in Shenyang, China, who were diagnosed with dementia between January 2007 and December 2012 Control: matched for every case via the medical reports archival system	Medical records from Chinese database of inhabitants of Shenyang	Medical record of prior GA	Dementia (DSM-IV) AD (DSM-IV)	Did not included lag time; matched for sex and age (within 1 year).	Cases		OR
							Dementia	577	0.81 (0.71–1.09)
							AD	485	0.89 (0.61–1.01)
							Controls		
							Dementia	577	1.00
							AD	485	1.00
Chen, 2014-2 [25] Taiwan	NCC	LHID (a subset of the NHIRD). Age ≥ 50 Case: newly diagnosed from 2005 to 2009 Control: 4-fold frequency matched	Medical records from the LHID	Record of endotracheal tube intubation GA	Dementia (clinical diagnosis using ICD-9-CM)	Did not included lag time; matched randomly by age (every 5 years of age), sex, and index year. Adjusted for age, sex, depression, diabetes mellitus, hypertension, stroke, and atherosclerosis.	Dementia group	5345	OR 1.34 (1.25–1.44)
							Control group	21380	1.00
Sprung, 2013 [59] Minnesota, USA	NCC	From residents of Olmsted County using Rochester Epidemiology Project (REP). Case: diagnosed between 1985 and 1994 Control: matched for each case	Medical records from the REP	Medical record of exposure to GA between age 45 and the index date	Dementia (DSM-IV) AD (DSM-IV, NINCDS-ADRDA)	Did not included lag time; matched randomly by sex and age (within 1 year).	Cases		OR (dementia/AD)
							Dementia	877	0.89 (0.73–1.10)
							AD	732	0.88 (0.71–1.11)
							Controls		
							Dementia	877	1.00
							AD	732	1.00
Bufill, 2009 [61] Spain	NCC	From subjects in COGMANLLEU study (belonging to the basic health care area of Manlleu). Age ≥ 80	Interview with participants and their relatives or caregivers	Self- or surrogate-reported	AD (DSM-IV, NINCDS-ADRDA)	Did not included lag time; matched for age and gender. Adjusted for age.	Cases	51	OR 3.22 (1.03–10.09)
							Controls	49	1.00
Yip, 2006 [28] England and Wales, UK	NCC	From Cognitive Function and Ageing Study (CFAS). Age ≥ 65 Case/control defined based on two times of interview	Interview with participant	Self-reported exposure to GA	Dementia (AGECAT algorithm)	Did not included lag time; adjusted for age, sex, education, and social class.	Cases: Dementia at wave 2/3	133/142	OR (wave 2/3/both) 0.7 (0.4–1.1) 0.6 (0.3–1.0) 0.6 (0.4–0.9)
							Controls: Wave 2/3	2453/1347	1.0 1.0 1.0
Harmanci, 2003 [14] Turkey	CC	Randomly selected from population registries (records of the Muhtars' list). Age ≥ 70 Case: probable AD patients Control: cognitively normal individuals identified by neurologic examination.	Interview with proxy informants	Surrogate-reported history of GA	AD (probable AD by NINCDS-ADRDA)	Did not included lag time; adjusted for level of education, use of electricity for residential heating, and occupational group.	Cases	57	OR 1.2 (0.58–2.48)
							Controls	127	1.0
Gasparini, 2002 [29] Italy	CC	Recruited at the Department of Neurological Sciences of “La Sapienza” University of Rome, who were treated between January 1990 and June 1997. Each case was matched for 4 controls (2 PD and 2 other disease)	Hospital records	Hospital record of exposure to GA in the 1-year and 5-year periods prior to onset of neurological disease.	AD (probable AD by NINCDS-ADRDA)	Did not included lag time; matched for sex, age (within 3 years), and geographical area of residence.	Cases	115	Calculated OR 1.03 (0.60–1.75)
							Controls (PD)	230	1.00
							Controls (others)	230	
Tyas, 2001 [60] Canada	CC	Randomly sampled from a list provided by the provincial health insurance plan. Age ≥ 65	Interview and questionnaire	Self-reported exposure to GA	AD (probable or possible AD by NINCDS-ADRDA)	Did not included lag time; adjusted for age, sex, education.	Cases	36	RR 0.61 (0.22–1.63)
							Controls	658	1.00
Bohnen, 1994 [10] Minnesota, USA	CC	Case: selected from patients with AD developed between 1975 and 1984 in Olmsted County by reviewing medical records Control: matched for each AD case from Olmsted County Mayo Clinic patients	Medical records	Anesthesia records for GA	AD (clinical diagnosis using their own preselected specific criteria)	Did not included lag time; matched for age, sex.	Cases	252	OR 1.28 (0.82–2.00)
							Controls	252	1.00
CHSA, 1994 [57] Canada	CC	Recruited from both the community and institutions in Canada. Age ≥ 65	Risk factor questionnaires completed by proxy respondents	Surrogate-reported exposure to GA	AD (probable AD by NINCDS-ADRDA)	Did not included lag time; frequency matching by study center, residence in community or institution, and age group Adjusted for age, sex, residence, and education	Cases	258	OR 1.07 (0.60–1.90)
							Controls	535	1.00
Li, 1992 [15] China	CC	Cases: Clinically diagnosed AD inpatients or outpatients from 1988 to 1989. Controls: Selected from the neighborhoods of the matched cases.	Direct interview using a structured and standardized questionnaire with surrogate informant.	Surrogate-reported history of GA	AD (probable AD by NINCDS-ADRDA, ICD-10)	Did not included lag time; Matched by age (within 3 years) and sex.	Cases	70	OR 0.62 (0.20–1.86)
							Controls	140	1.00
Kokmen, 1991 [11] Minnesota, USA	CC	Cases: Rochester, Minnesota, residents with onset of AD between 1960 and 1974 using the existing medical records resource. Controls: matched for each case by searching the registration system at Mayo Clinic.	Entire community medical records.	Medical record of prior GA	AD (clinical diagnosis by reviewing clinical and postmortem data)	Did not included lag time; matched by age (within years), sex, and duration of community medical record.	Cases	415	OR 0.86 (0.60–1.21)
							Controls	415	1.00
Graves, 1990 [56] Washington, USA	CC	Cases: patients living in Washington state who were diagnosed with AD between January 1980 and June 1985. Controls: friend, relative or surrogate of the cases.	Interview with surrogate respondents	Surrogate-reported history of surgery with GA	AD (DSM-III, NINCDS-ADRDA)	Did not included lag time; matched by sex and age (within 10 years). Adjusted for age in the reference year.	Cases	130	OR 1.21 (0.54–2.73)
							Controls	130	1.00
Broe, 1990 [58] Australia	CC	Cases: from consecutive new referrals to dementia clinics in Sydney by general practitioners (GPs). Controls: a person matched for each case from same GP's files.	Interview with the informants of the cases and controls	Surrogate-reported exposure to GA	AD (probable or possible AD by NINCDS-ADRDA)	Did not included lag time; matched for sex and age within 2 years. Matched pairs odds ratio was calculated.	Cases	170	OR 0.95 (0.50–1.81)
							Controls	170	1.00
Amaducci, 1986 [12] Italy	CC	Cases: Patients admitted to the neurology departments of the seven centers between 1982 and 1983. Controls: 1 hospital (from same hospital) and 1 population control (neighbor, a friend, or an acquaintance) was identified for each case	Interview with a surrogate respondent.	Surrogate-reported exposure to GA	AD (clinical diagnosis using their own criteria)	Did not included lag time; Matched for age (within 3 years), sex, and region of residence. Matched-pair analysis were used.	Cases	116	Calculated OR 1.00 (0.38–2.66)
							Controls (hospital)	116	1.00
							Controls (population)	97	
Heyman, 1984 [13] USA	CC	Cases: participants in a comprehensive clinical, genetic, and epidemiological study of AD at Duke University Medical Center. Control: 2 matched subjects selected by the telephone sampling technique of random-digit dialing.	Structured interview with a close family member.	Surrogate-reported history of surgery with GA	AD (clinical diagnosis using their own diagnostic procedure)	Did not included lag time; matched for sex, race, 5-year age interval (50-54, 55-59, etc.), and residential area.	Cases	40	Calculated OR 0.63 (0.20–1.96)
							Controls	80	1.00

Abbreviations. *N*: number of subjects; CI: confidence interval; HR: hazard ratio; OR: odds ratio; RR: relative risk; AD: Alzheimer's disease; GA: general anesthesia; PC: prospective cohort study; RC: retrospective cohort study; NCC: nested case-control study; CC: case-control study; ICD-10: International Classification of Diseases, 10th Revision; ICD-9-CM: International Classification of Diseases, 9th Revision, Clinical Modification; DSM-III: Diagnostic and Statistical Manual of Mental Disorders, 3rd Edition; DSM-IV: Diagnostic and Statistical Manual of Mental Disorders, 4th Edition; NINCDS-ADRDA: National Institutes of Neurological and Communicative Disorders and Stroke-Alzheimer's Disease and Related Disorders; AGECAT: Automated Geriatric Examination for Computer Assisted Taxonomy; NHIS-NSC: National Health Insurance Service-National Sample Cohort; LHID: Longitudinal Health Insurance Database; NHIRD: National Health Insurance Research Database; AOK: Allgemeine Ortskrankenkasse; CDR: Clinical Data Repository; CABG: coronary artery bypass graft; PTCA: percutaneous transluminal coronary angioplasty; PD: Parkinson's disease; CHSA: The Canadian Study of Health and Aging.

**Table 2:** Quality assessment of included studies using the Risk of Bias Assessment Tool for Nonrandomized Studies (RoBANS).

	Risk of bias					
	Selection of participants	Confounding variables	Measurement of exposure	Blinding of outcome assessments	Incomplete outcome data	Selective outcome reporting
Cohort study						
Kim, 2018 [27]	Low	Low	Low	Low	Low	Unclear
Teipel, 2018 [31]	Low	Low	Low	Low	Low	Unclear
Aiello Bowles, 2016 [30]	Low	Low	Unclear	Low	Low	Unclear
Chen, 2014-1 [26]	Low	Low	Low	Low	Low	Unclear
Zuo, 2010 [8]	Low	High	Low	Low	Low	Unclear
Lee, 2005 [9]	Low	Low	Low	Low	Low	Unclear
Case-control study						
Strand, 2019 [62]	Low	Low	Low	Low	Low	Unclear
Huang, 2018 [63]	Low	Low	Low	Low	Low	Unclear
Chen, 2014-2 [25]	Low	Low	Low	Low	Low	Unclear
Sprung, 2013 [59]	Low	Low	Low	Low	Low	Unclear
Bufill, 2009 [61]	Low	Low	Unclear	Low	Low	Unclear
Yip,2006 [28]	Low	Low	Unclear	Low	Low	Unclear
Harmanci, 2003 [14]	Low	Low	Unclear	Low	Low	Unclear
Gasparini, 2002 [29]	Unclear	Low	Low	Low	Low	Unclear
Tyas, 2001 [60]	Low	Low	Unclear	Low	Low	Unclear
Bohnen 1994 [10]	Low	Low	Low	Low	Low	Unclear
CHSA, 1994 [57]	Low	Low	Unclear	Low	Low	Unclear
Li, 1992 [15]	Low	Low	Unclear	Low	Low	Unclear
Kokmen, 1991 [11]	Low	Low	Low	Low	Low	Unclear
Graves, 1990 [56]	Low	Low	Unclear	Low	Low	Unclear
Broe, 1990 [58]	Low	Low	Unclear	Low	Low	Unclear
Amaducci, 1986 [12]	Low	Low	Unclear	Low	Low	Unclear
Heyman, 1984 [13]	Low	Low	Unclear	Low	Low	Unclear

**Table 3:** Summary estimates, heterogeneity, and publication bias for meta-analyses of overall studies and subgroups.

Characteristics	Summary estimates			Heterogeneity		Publication bias		Trim and fill
	No. of studies	Pooled ES (95% CI)	95% PI	*p*	*I* ^2^ (%)	*p*	Coef (95% CI)	Pooled ES (95% CI)
Total	23	1.11 (1.06–1.15)	0.98-1.21	<0.001	79.4	0.096	-0.91 (-1.99–0.24)	1.09 (0.94–1.27)
Design								
Cohort	6	1.11 (1.06–1.16)	0.93-1.27	<0.001	88.5			
Case-control	17	1.15 (1.03–1.17)	0.81-1.20	<0.001	74.8	0.088	-1.02 (-2.22–0.17)	1.07 (0.87–1.32)
Region								
America	10	0.83 (0.72–0.94)	0.48-1.1	0.354	9.6	0.626	0.49 (-1.73–2.71)	
Australia	1	0.95 (0.50–1.81)						
Asia	5	1.23 (1.17–1.28)	0.98-1.41	<0.001	87.5			
Europe	7	0.96 (0.88–1.05)	0.68-1.22	0.055	51.3			
Exposure assessment								
Medical record	12	1.15 (1.10–1.19)	1.01-1.25	<0.001	84.1	0.442	-0.89 (-3.38–1.59)	
Self or surrogate-reported	11	0.73 (0.59–0.87)	0.28-1.08	0.777	0.0	0.155	1.11 (-0.51–2.72)	
Dementia definition								
Alzheimer's disease	17	0.86 (0.76–0.95)	0.58-1.07	0.703	0.0	0.260	0.72 (-0.60–2.04)	
Dementia	6	1.18 (1.13–1.23)	0.99-1.28	<0.001	90.6			
Case ascertainment								
Standard criteria^a^	13	0.82 (0.72–0.92)	0.73-1.07	0.478	0.0	0.147	1.09 (-0.45–2.63)	
Clinical diagnosis	10	1.18 (1.13–1.23)	1.08-1.39	<0.001	81.2	0.493	-0.75 (-3.17–1.67)	

Abbreviations. Pooled ES: pooled estimates; PI: prediction interval; Coef: coefficient. ^a^Standard criteria represent the diagnosis for Alzheimer's disease or dementia using object criteria or algorithm such as Diagnostic and Statistical Manual of Mental Disorders (DSM), National Institutes of Neurological and Communicative Disorders and Stroke-Alzheimer's Disease and Related Disorders (NINCDS-ADRDA), and Automated Geriatric Examination for Computer Assisted Taxonomy (AGECAT) algorithm.

**Table 4:** Meta-regression for overall studies.

Category	Coef	95% confidence interval	*p* value
Study design	-0.00	-0.13–0.13	0.944
Region	0.02	-0.09–0.13	0.755
Exposure assessment	-0.03	-0.40–0.34	0.866
Case ascertainment	-0.00	-0.34–0.34	0.992
Outcome definition	-0.08	-0.53–0.36	0.694
Year of publication	0.00	-0.02–0.02	0.899
Constants	-2.09	-39.28–35.09	0.906

Abbreviation. Coef: coefficient.

## Data Availability

The study data supporting this systematic review and meta-analysis are from previously reported studies and datasets, which have been cited. The processed data are available in the article.

## Supplementary Materials

Supplementary Materials Appendix S1. Search terms used in literature search.

## References

[1] Sieber F. E., Barnett S. R. (2011). Preventing postoperative complications in the elderly. *Anesthesiology Clinics*.

[2] LoGiudice D., Watson R. (2014). Dementia in older people: an update. *Internal Medicine Journal*.

[3] GBD 2015 Disease and Injury Incidence and Prevalence Collaborators (2016). Global, regional, and national incidence, prevalence, and years lived with disability for 310 diseases and injuries, 1990–2015: a systematic analysis for the Global Burden of Disease Study 2015. *The Lancet*.

[4] Prince M., Bryce R., Albanese E., Wimo A., Ribeiro W., Ferri C. P. (2013). The global prevalence of dementia: a systematic review and metaanalysis. *Alzheimers Dementia*.

[5] Park J. H., Eum J. H., Bold B., Cheong H. K. (2013). Burden of disease due to dementia in the elderly population of Korea: present and future. *BMC Public Health*.

[6] Alzheimer's Association (2018). 2018 Alzheimer's disease facts and figures. *Alzheimers Dementia*.

[7] McKhann G. M., Knopman D. S., Chertkow H. (2011). The diagnosis of dementia due to Alzheimer's disease: recommendations from the national institute on aging-Alzheimer's association workgroups on diagnostic guidelines for Alzheimer's disease. *Alzheimers Dementia*.

[8] Zuo C., Zuo Z. (2010). Spine surgery under general anesthesia may not increase the risk of alzheimer's disease. *Dementia and Geriatric Cognitive Disorders*.

[9] Lee T. A., Wolozin B., Weiss K. B., Bednar M. M. (2005). Assessment of the emergence of Alzheimer's disease following coronary artery bypass graft surgery or percutaneous transluminal coronary angioplasty. *Journal of Alzheimer's Disease*.

[10] Bohnen N. I., Warner M. A., Kokmen E., Beard C. M., Kurland L. T. (1994). Alzheimer's disease and cumulative exposure to anesthesia: a case-control study. *Journal of the American Geriatrics Society*.

[11] Kokmen E., Beard C. M., Chandra V., Offord K. P., Schoenberg B. S., Ballard D. J. (1991). Clinical risk factors for Alzheimer's disease: a population-based case-control study. *Neurology*.

[12] Amaducci L. A., Fratiglioni L., Rocca W. A. (1986). Risk factors for clinically diagnosed Alzheimer's disease: a case-control study of an italian population. *Neurology*.

[13] Heyman A., Wilkinson W. E., Stafford J. A., Helms M. J., Sigmon A. H., Weinberg T. (1984). Alzheimer's disease: a study of epidemiological aspects. *Annals of Neurology*.

[14] Harmanci H., Emre M., Gurvit H. (2003). Risk factors for Alzheimer disease: a population-based case-control study in Istanbul, Turkey. *Alzheimer Disease & Associated Disorders*.

[15] Li G., Shen Y. C., Li Y. T., Chen C. H., Zhau Y. W., Silverman J. M. (1992). A case-control study of Alzheimer's disease in China. *Neurology*.

[16] Marques A., Lapa T. (2018). Anesthesia and alzheimer disease - current perceptions. *Revista Brasileira de Anestesiologia*.

[17] Hussain M., Berger M., Eckenhoff R. G., Seitz D. P. (2014). General anesthetic and the risk of dementia in elderly patients: current insights. *Clinical Interventions in Aging*.

[18] Bittner E. A., Yue Y., Xie Z. (2011). Brief review: anesthetic neurotoxicity in the elderly, cognitive dysfunction and Alzheimer's disease. *Canadian Journal of Anaesthesia*.

[19] Breteler M. M. B., van Duijn C. M., Chandra V. (1991). Medical history and the risk of Alzheimer's disease: a collaborative re-analysis of case-control Studies. *International Journal of Epidemiology*.

[20] Jiang J., Dong Y., Huang W., Bao M. (2017). General anesthesia exposure and risk of dementia: a meta-analysis of epidemiological studies. *Oncotarget*.

[21] Seitz D. P., Shah P. S., Herrmann N., Beyene J., Siddiqui N. (2011). Exposure to general anesthesia and risk of Alzheimer's disease: a systematic review and meta-analysis. *BMC Geriatrics*.

[22] Choi G. J., Kang H., Baek C. W., Jung Y. H., Kim J. W., Woo Y. C. (2017). Relationship between general anesthesia and alzheimer disease: a protocol for a systematic review and meta-analysis. *Medicine*.

[23] Stroup D. F., Berlin J. A., Morton S. C. (2000). Meta-analysis of observational studies in epidemiology: a proposal for reporting. Meta-analysis of observational studies in epidemiology (MOOSE) group. *JAMA*.

[24] Liberati A., Altman D. G., Tetzlaff J. (2009). The PRISMA statement for reporting systematic reviews and meta-analyses of studies that evaluate healthcare interventions: explanation and elaboration. *BMJ*.

[25] Chen C. W., Lin C. C., Chen K. B., Kuo Y. C., Li C. Y., Chung C. J. (2014). Increased risk of dementia in people with previous exposure to general anesthesia: a nationwide population-based case-control study. *Alzheimers & Dementia*.

[26] Chen P. L., Yang C. W., Tseng Y. K. (2014). Risk of dementia after anaesthesia and surgery. *British Journal of Psychiatry*.

[27] Kim C. T., Myung W., Lewis M. (2018). Exposure to general anesthesia and risk of dementia: a nationwide population-based cohort study. *Journal of Alzheimer's Disease*.

[28] Yip A. G., Brayne C., Matthews F. E., MRC Cognitive Function and Ageing Study (2006). Risk factors for incident dementia in England and Wales: the Medical Research Council Cognitive Function and Ageing Study. A population-based nested case-control study. *Age and Ageing*.

[29] Gasparini M., Vanacore N., Schiaffini C. (2002). A case-control study on Alzheimer's disease and exposure to anesthesia. *Neurological Sciences*.

[30] Aiello Bowles E. J., Larson E. B., Pong R. P. (2016). Anesthesia exposure and risk of dementia and Alzheimer's disease: a prospective study. *Journal of the American Geriatrics Society*.

[31] Teipel S. J., Fritze T., Ellenrieder M., Haenisch B., Mittelmeier W., Doblhammer G. (2018). Association of joint replacement surgery with incident dementia diagnosis in German claims data. *International Psychogeriatrics*.

[32] Kim S. Y., Park J. E., Lee Y. J. (2013). Testing a tool for assessing the risk of bias for nonrandomized studies showed moderate reliability and promising validity. *Journal of Clinical Epidemiology*.

[33] Higgins J. P., Thompson S. G. (2002). Quantifying heterogeneity in a meta-analysis. *Statistics in Medicine*.

[34] Higgins J. P., Thompson S. G., Deeks J. J., Altman D. G. (2003). Measuring inconsistency in meta-analyses. *BMJ*.

[35] Egger M., Smith G. D. (1998). Bias in location and selection of studies. *BMJ*.

[36] Plassman B. L., Langa K. M., Finlayson E. V. A., Rogers M. A. M. (2009). P3-153: Surgery using general anesthesia and risk of dementia in the aging, demographics and memory study. *Alzheimers Dementia*.

[37] Arora S. S., Gooch J. L., Garcia P. S. (2014). Postoperative cognitive dysfunction, Alzheimer's disease, and anesthesia. *International Journal of Neuroscience*.

[38] Jiang T., Yu J. T., Tian Y., Tan L. (2013). Epidemiology and etiology of alzheimer's disease: from genetic to non-genetic factors. *Current Alzheimer Research*.

[39] Mathews S. B., Arnold S. E., Epperson C. N. (2014). Hospitalization and cognitive decline: can the nature of the relationship be deciphered?. *American Journal of Geriatric Psychiatry*.

[40] Seitz D. P., Reimer C. L., Siddiqui N. (2013). A review of epidemiological evidence for general anesthesia as a risk factor for Alzheimer's disease. *Progress in Neuro-Psychopharmacology & Biological Psychiatry*.

[41] Tanner C. M., Goldman S. M., Ross G. W., Grate S. J. (2014). The disease intersection of susceptibility and exposure: chemical exposures and neurodegenerative disease risk. *Alzheimers Dementia*.

[42] Deiner S., Silverstein J. H. (2009). Postoperative delirium and cognitive dysfunction. *British Journal of Anaesthesia*.

[43] Evered L. (2013). Dissecting the possible influences of anesthesia and surgery on Alzheimer's disease. *Neurodegenerative Disease Management*.

[44] Vanderweyde T., Bednar M. M., Forman S. A., Wolozin B. (2010). Iatrogenic risk factors for Alzheimer's disease: surgery and anesthesia. *Journal of Alzheimer's Disease*.

[45] Ballard C., Clack H., Green D. (2007). Postoperative cognitive decline, dementia and anaesthesia. *British Journal of Hospital Medicine*.

[46] Harris R. A., Eger E. I. (2008). Alzheimer's disease and anesthesia: out of body, out of mind…Or not?. *Annals of Neurology*.

[47] Scott D. A., Silbert B. S., Evered L. A. (2013). Anesthesia and Alzheimer's disease: time to wake up!. *International Psychogeriatrics*.

[48] Baranov D., Bickler P. E., Crosby G. J. (2009). Consensus statement: first international workshop on anesthetics and Alzheimer's disease. *Anesthesia & Analgesia*.

[49] Barnes D. E., Covinsky K. E., Whitmer R. A., Kuller L. H., Lopez O. L., Yaffe K. (2009). Predicting risk of dementia in older adults: the late-life dementia risk index. *Neurology*.

[50] Bohnen N., Warner M. A., Kokmen E., Kurland L. T. (1994). Early and midlife exposure to anesthesia and age of onset of Alzheimer's disease. *International Journal of Neuroscience*.

[51] de Oliveira F. F., Bertolucci P. H., Chen E. S., Smith M. C. (2014). Assessment of risk factors for earlier onset of sporadic Alzheimer's disease dementia. *Neurology India*.

[52] Fischer P., Wallner H., Jungwirth S. (2007). Cumulative exposure to general anesthesias and cognitive dysfunction at age 75 in the Vienna transdanube aging "VITA" study. *The Journal of Neuropsychiatry and Clinical Neurosciences*.

[53] Ritchie K., Carriere I., Ritchie C. W., Berr C., Artero S., Ancelin M. L. (2010). Designing prevention programmes to reduce incidence of dementia: prospective cohort study of modifiable risk factors. *BMJ*.

[54] French L. R., Schuman L. M., Mortimer J. A., Hutton J. T., Boatman R. A., Christians B. (1985). A case-control study of dementia of the alzheimer type. *American Journal of Epidemiology*.

[55] Tsuda Y., Yasunaga H., Horiguchi H., Ogawa S., Kawano H., Tanaka S. (2015). Association between dementia and postoperative complications after hip fracture surgery in the elderly: analysis of 87,654 patients using a national administrative database. *Archives of Orthopaedic and Trauma Surgery*.

[56] Graves A. B., White E., Koepsell T. D. (1990). A case-control study of Alzheimer's disease. *Annals of Neurology*.

[57] The Canadian Study of Health and Aging (1994). The Canadian study of health and aging: risk factors for alzheimer's disease in Canada. *Neurology*.

[58] Broe G. A., Henderson A. S., Creasey H. (1990). A case-control study of Alzheimer's disease in Australia. *Neurology*.

[59] Sprung J., Jankowski C. J., Roberts R. O. (2013). Anesthesia and incident dementia: a population-based, nested, case-control study. *Mayo Clinic Proceedings*.

[60] Tyas S. L., Manfreda J., Strain L. A., Montgomery P. R. (2001). Risk factors for Alzheimer's disease: a population-based, longitudinal study in Manitoba, Canada. *International Journal of Epidemiology*.

[61] Bufill E., Bartés A., Moral A. (2009). Genetic and environmental factors that may influence in the senile form of Alzheimer's disease: nested case control studies. *Neurología*.

[62] Strand A. K., Nyqvist F., Ekdahl A., Wingren G., Eintrei C. (2019). Is there a relationship between anaesthesia and dementia?. *Acta Anaesthesiologica Scandinavica*.

[63] Huang Z., Wang N., Suo L., Wang M. (2018). Single or multiple encounters of general anaesthesia do not cause any cognitive dysfunction: findings from a retrospective population-based, case-control study. *Pakistan Journal of Pharmaceutical Sciences*.

[64] Zuo Z. (2012). Are volatile anesthetics neuroprotective or neurotoxic?. *Medical Gas Research*.

[65] Custodio N., Montesinos R., Lira D., Herrera-Pérez E., Bardales Y., Valeriano-Lorenzo L. (2017). Mixed dementia: a review of the evidence. *Dementia & Neuropsychologia*.

[66] Zhou Z. B., Meng L., Gelb A. W., Lee R., Huang W. Q. (2016). Cerebral ischemia during surgery: an overview. *Journal of Biomedical Research*.

[67] Lucchetta R. C., da Mata B. P. M., Mastroianni P. C. (2018). Association between development of dementia and use of benzodiazepines: a systematic review and meta-analysis. *Pharmacotherapy*.

[68] Cerejeira J., Batista P., Nogueira V., Vaz-Serra A., Mukaetova-Ladinska E. B. (2013). The stress response to surgery and postoperative delirium: evidence of hypothalamic–pituitary–adrenal axis hyperresponsiveness and decreased suppression of the gh/igf-1 axis. *Journal of Geriatric Psychiatry and Neurology*.

[69] Kimchi E. Y., Hshieh T. T., Guo R. (2017). Consensus approaches to identify incident dementia in cohort studies: systematic review and approach in the successful aging after elective surgery study. *Journal of the American Medical Directors Association*.

[70] Bai J., Zhang P., Liang X., Wu Z., Wang J., Liang Y. (2018). Association between dementia and mortality in the elderly patients undergoing hip fracture surgery: a meta-analysis. *Journal of Orthopaedic Surgery and Research*.

[71] Lee S. J., Jung S. H., Lee S.-U., Lim J.-Y., Yoon K.-S., Lee S. Y. (2020). Postoperative delirium after hip surgery is a potential risk factor for incident dementia: a systematic review and meta-analysis of prospective studies. *Archives of Gerontology and Geriatrics*.

[72] Diaz-Gil A., Brooke J., Kozlowska O., Pendlebury S., Jackson D. (2018). Care needs of people with dementia in the peri-operative environment: a systematic review. *Dementia*.

