# Supplementary material for: Relationship between Surgery under General Anesthesia and the Development of Dementia: A Systematic Review and Meta-Analysis
Source: Biomed Res Int. 2020 Apr 2;2020:3234013. doi: 10.1155/2020/3234013 (PMC7165327; doi:10.1155/2020/3234013)
Supplement: Supplementary Materials — Appendix S1. Search terms used in literature search. [file 3234013.f1.docx]

**Appendix 1. Search terms used in literature search**

***Search terms for MEDLINE***

1. Exp dementia/

2. Dementia.mp.

3. Amentia.mp.

4. Alzheimer.mp.

5. or/1-4

6. exp anesthesia/

7. exp anesthetics/

8. an?esthesia.mp.

9. anesth*.mp.

10. anaesth*.mp.

11. Or/6-10

12. 5 and 11

13. Case report.tw.

14. Letter.pt.

15. Historical article.pt.

16. Review.pt.

17. or/13-16

18. 12 not 17

***Search terms for EMBASE***

1. 'dementia'/exp

2. Dementia

3. Amentia

4. 'alzheimer disease'

5. #1 OR #2 OR #3 OR #4

6. 'anesthesia'/exp

7. 'anesthetic agent'/exp

8. anaesthesia

9. anaesthetic.mp.

10. #6 OR #7 OR #8 OR #9

11. #5 AND #10

12. 'case study'/exp

13. 'case report'/exp

14. 'abstract report'/exp

15. 'letter'/exp

16. #12 OR #13 OR #14 OR #15

17. #11 NOT #16
